# Deep Learning for Diagnostic Binary Classification of Multiple-Lesion Skin Diseases

**DOI:** 10.3389/fmed.2020.574329

**Published:** 2020-09-22

**Authors:** Kenneth Thomsen, Anja Liljedahl Christensen, Lars Iversen, Hans Bredsted Lomholt, Ole Winther

**Affiliations:** ^1^Department of Dermatology and Venereology, Aarhus University Hospital, Aarhus, Denmark; ^2^Department of Applied Mathematics and Computer Science, Technical University of Denmark, Lyngby, Denmark; ^3^Clinical Institute, Aalborg University, Aalborg, Denmark; ^4^Center for Genomic Medicine, Rigshospitalet, Copenhagen University Hospital, Copenhagen, Denmark; ^5^Department of Biology, Bioinformatics Centre, University of Copenhagen, Copenhagen, Denmark

**Keywords:** deep neural network (DNN), dermatology, skin disease, acne, rosacea, psoriasis, cutaneous T cell lymphoma (CTCL), ezcema

## Abstract

**Background:** Diagnosis of skin diseases is often challenging and computer-aided diagnostic tools are urgently needed to underpin decision making.

**Objective:** To develop a convolutional neural network model to classify clinically relevant selected multiple-lesion skin diseases, this in accordance to the STARD guidelines.

**Methods:** This was an image-based retrospective study using multi-task learning for binary classification. A VGG-16 model was trained on 16,543 non-standardized images. Image data was distributed in training set (80%), validation set (10%), and test set (10%). All images were collected from a clinical database of a Danish population attending one dermatological department. Included was patients categorized with ICD-10 codes related to acne, rosacea, psoriasis, eczema, and cutaneous t-cell lymphoma.

**Results:** Acne was distinguished from rosacea with a sensitivity of 85.42% CI 72.24–93.93% and a specificity of 89.53% CI 83.97–93.68%, cutaneous t-cell lymphoma was distinguished from eczema with a sensitivity of 74.29% CI 67.82–80.05% and a specificity of 84.09% CI 80.83–86.99%, and psoriasis from eczema with a sensitivity of 81.79% CI 78.51–84.76% and a specificity of 73.57% CI 69.76–77.13%. All results were based on the test set.

**Conclusion:** The performance rates reported were equal or superior to those reported for general practitioners with dermatological training, indicating that computer-aided diagnostic models based on convolutional neural network may potentially be employed for diagnosing multiple-lesion skin diseases.

## Introduction

Skin diseases rank fourth among non-fatal diseases with respect to global burden (1) and are, estimated to account for 12–20% of general practitioner (GP) consultations (2, 3). With more than 1,500 different dermatological diagnoses (4), differential diagnosing can be very challenging. GP diagnostic accuracy in dermatological disease has been estimated to fall in the 48–77% range (5). For GP's distinguishing between the two morphologically similar and common papulo-pustular skin diseases of acne and rosacea, and between the two common scaly erythematous diseases of psoriasis and eczema can be a challenge. Furthermore, cutaneous t-cell lymphoma (CTCL) is a rare malignant disease of the skin that is often difficult to distinguish from eczematous disease, even for trained dermatologists (6). Low diagnostic accuracy in primary healthcare combined with reports of a growing shortage of dermatologists in rural parts of the US (7) carry a risk of untimely treatment and triaging.

Computer-aided diagnostic (CAD) models based on convolutional neural network (CNN) have been developed with promising results for distinguishing what is typically single-lesion skin diseases, such as malignant melanoma, squamous cell carcinoma, or nail dystrophies (8–11). CAD models developed for these diseases are often trained by standardized imagery such as dermatoscopic images (8). Reports on CAD models for multiple-lesion skin diseases are few and have shown more moderate performance rates (8). A Google-associated research team published results on a combined image and text classifier for dermatology (12). This model achieved a 67–75% sensitivity in diagnosing multiple-lesion skin disease (including acne, eczema, and psoriasis). Recently Wu et al. did show an impressive 95% overall diagnostic accuracy in classifying atopic dermatitis, eczema and psoriasis on selected image material (13). Studies comparing the accuracy of CAD models to clinicians are generally based on image classification equivalent to retrospective analysis, though some head to head studies were conducted with prospective collected image material (12, 14, 15).

Machine learning models, broadly characterized as CNNs, have proven their merits in image classification (16). A CNN is a layered statistical model using two-dimensional convolutions, element-wise non-linearities, and local pooling operations in the convolutional layers. The input to a convolutional layer is a representation of the data from the previous layer. The first layer is the original color image of size “height times width times three” (for the three-color channels). The produced data representations are called feature maps. These maps should ideally capture some property of the original data relevant for the classification. After a number of convolutional layers, the final feature maps are collapsed into a vector that is fed into a number of fully connected layers. The final output layer uses a so-called softmax function to calculate the model's estimation of the class probabilities.

Open source CNN models are available both for training from scratch and for transfer learning (modifying parts of an extant model for a new task). These models are often tested on the ImageNet dataset of more than 1 million labeled high-resolution images, in the yearly ImageNet Large Scale Visual Recognition Challenge (ILSVRC). The VGG-16 model is based on the architecture developed by the Oxfords Visual Geometry Group (VGG) (17) and achieved top performance in the ILSVRC 2014.

The primary aim of this feasibility study was to investigate a diagnostic tool to assist primarily GPs in distinguishing between patients with common and rare multiple-lesion skin diseases that often have a similar clinical presentation, this in accordance to the STARD guidelines for reporting on diagnostic accuracy (18). We aimed to achieve a differential diagnostic accuracy equal to or above the 48–77% reported for GPs (5, 19). We focused on five multiple-lesion skin diseases and on non-standardized imagery to accommodate the paucity in the scientific literature (8), and—more importantly—to imitate the real-life clinical settings of primary healthcare professionals, where multiple-lesion dermatological diseases are often encountered and access to a dermatoscope is limited (20, 21).

## Materials and Methods

### Dataset

A total of 19,641 images were provided from the local skin image database of the Department of Dermatology, Aarhus University Hospital (AUH), Denmark. The images were collected from 2,342 patients of a Danish population and therefore comprise mainly images of patients with Fitzpatrick skin type II and III, see Table 1 for the disease distribution of the image data and patients. The database was designed for clinical reference such as disease monitoring and plenum discussion, and therefore certain non-skin images were included. Non-skin images were mainly yellow patient identification slips and skin sensitizers related to contact eczema. The data set was cleansed by a simple CNN model trained on 200 skin and 200 non-skin images. This model was tested on 150 images of both skin and non-skin images and removed all non-skin images from the test set with an accuracy of 99%. The architecture is shown in Supplementary Figure 1. After cleansing, 16,453 only-skin images were included for further investigation. A sampling counting 208 random images from the data set showed that 3.7% of the images represented healthy skin. No further effort was made to remove healthy skin images from the data. All images were non-standardized photographs, in different resolutions, shot by a clinical photographer using a blue background or by a healthcare worker or by the patients themselves, the latter two with random background.

**Table 1:** Data distribution in disease category.

**Disease**	**Included**	**Excluded**	**Patients included**
	**images**	**images**	**in final dataset**
Psoriasis	6,545	1,052	790
Eczema/atopic dermatitis	5,350	977	870
CTCL	2,461	380	157
Acne	581	155	131
Rosacea	1,606	534	394
Total	16,543	3,098	2,342

*Data and patient distribution in disease categories after data cleansing, for details on data cleansing see Supplementary Table 1*.

All images were diagnosed by trained dermatologists from the AUH according to the International Classification of Diseases, 10th Version (ICD-10). For the ICD-10 codes included in each disease category, see the Supplementary Table 1. CTCL diagnosis were histologically verified.

**Figure 1:**
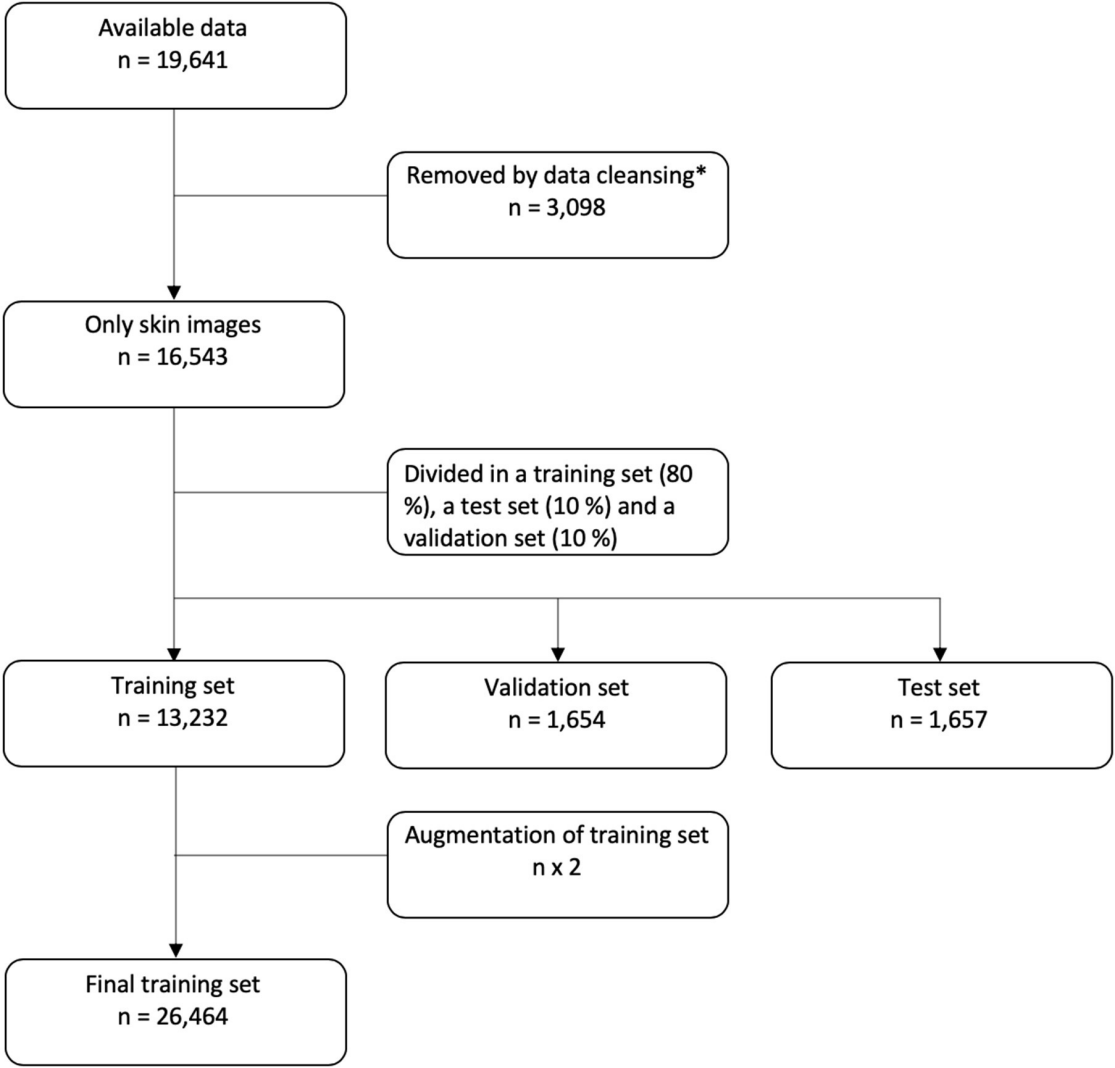
Flowchart of data. *For details on data cleansing, please see the Supplementary Figure 1.

In the final data set, 80% of the data were used for training of the CNN, 10% were used as a validation set, and 10% were saved as a test set. For patients with multiple images, all images were placed either in the training, validation or test set. So the same patient will not have images used for both training and testing. The flow of data is shown in Figure 1.

### Data Augmentation

Data augmentation of the training data set was applied randomly on each of the images to duplicate the samples by randomly either zooming in or out, flipping vertically or horizontally, rotating, or shifting.

### Ethics

This study was conducted in concordance with the European General Data Protection Regulation. All relevant governmental bodies were notified of the study and usages of the image database.

The study was approved by the data controller of the clinical image database according to §10 of the Danish privacy act.

The Regional Ethics Committee of the Central Denmark Region (case no. 177/2018) deemed the study as not being a health care project, and authorized the project to proceed without their approval.

The Danish Patient Safety Authority (case no. 31-1521-68) authorized the usage of the clinical image database without patient consent.

Finally, the study was registered in the Regional Research Study Registry of Central Denmark Region under the Danish Data Protection Agency (case no. 1-16-02-373-19).

### Pre-processing

Cropping of noise, i.e., clothes, background and jewelry, in the pictures was done by K-means clustering using the pixel hue values. Using K = 2 clusters, each image was segmented into a skin cluster and a non-skin cluster (22). An image-dependent ratio was used with lower bound of 0.1 and upper bound of 0.3 to avoid under and over cropping, respectively. Examples of image cropping can be found in Supplementary Figure 2. All models were tested on original, cropped, and balanced versions of the data.

### Tasks

Four binary CNN models were trained using data from all of the five diseases, afterwards model tests were conducted focusing on three separate binary tasks of all four binary models. This method is known as multi-task learning.

Task 1: classification of psoriasis vs. eczema; task 2: classification of acne vs. rosacea; and task 3: classification of CTCL (mainly images of mycosis fungoides, see Supplementary Table 1 of ICD-10 code distribution) vs. eczema.

The primary outcome was the sensitivity and the specificity of the best model in the binary classification task. The secondary outcome was to define the best model by the area under the curve (AUC) and accuracy.

### CNN Models

**Figure 2:**
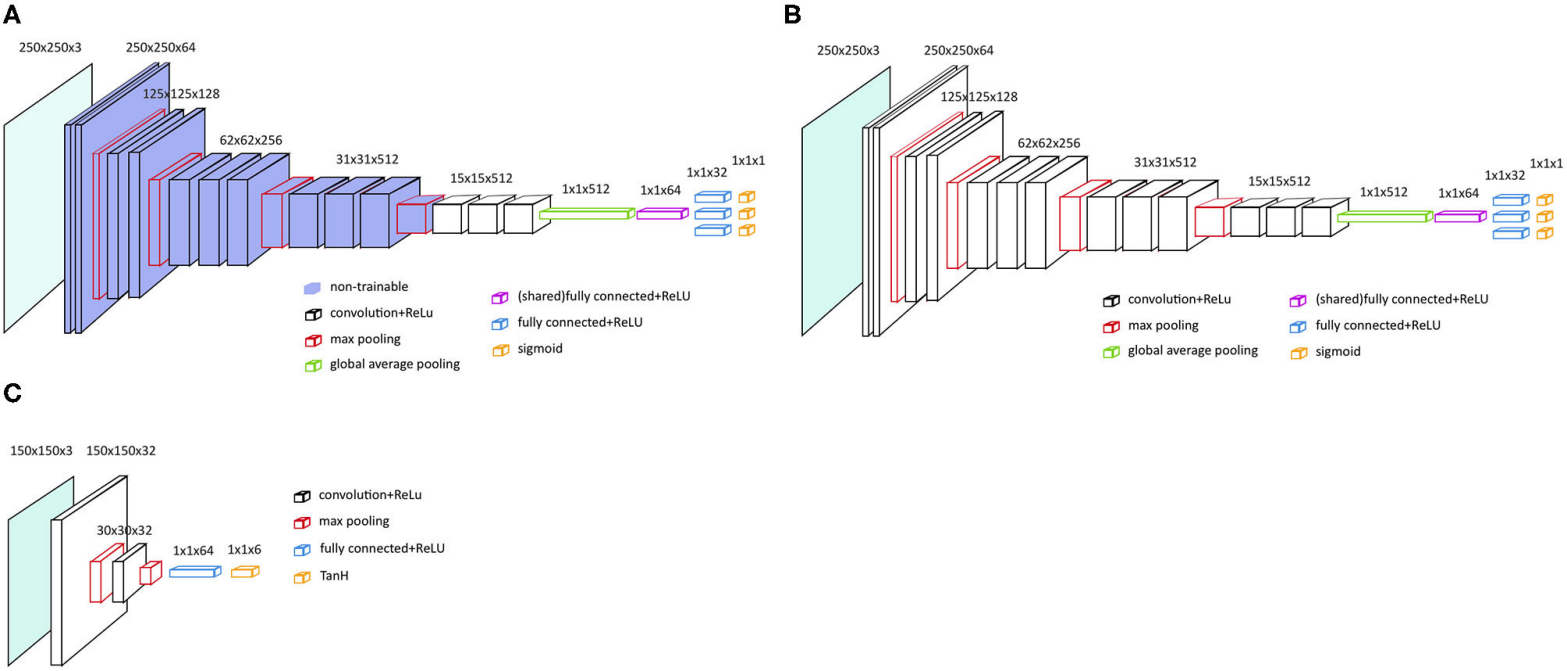
Main architecture of the VGG-16 models. **(A)** VGG-16 model with frozen and pre-trained layers, i.e., VGG-16P and VGG-16PS. **(B)** VGG-16 architecture with no frozen layers and no pre-trained layers, i.e. VGG-16N and VGG-16NS. **(C)** Localization network with two convolutional layers, as applied for VGG-16PS and VGG-16NS.

As a base model we choose VGG-16 with pre-trained parameters from the ILSVRC data set and no spatial transformer network (STN) (VGG-16P) (23). The fully connected layers and softmax layers were removed and replaced by new randomly initialized, fully connected layers and sigmoid layers, see Figure 2A. The pre-trained convolutional layers of the VGG-16 model were frozen.

To test if an even better performance could be achieved by training all the parameters from scratch, we also tested the VGG-16 with no pre-trained parameters and no STN (VGG-16N); see the architecture in Figure 2B.

To test if addition of a STN would increase the VGG-16 classification performance by assisting the VGG-16 in selecting the region of interest in an image (24), we developed two models similar to the two models described above, but added a STN; VGG-16 with pre-trained parameters and a STN (VGG-16PS) and VGG-16 with no pre-trained parameters and a STN (VGG-16NS).

Initial tests showed that the implementation of a STN was better with a localization network with two convolutional layers (Figure 2C).

See Supplementary Table 2 “hyperparameters and hardware details” for more on this matter.

### Data Presentation

In accordance with guidelines for developing and reporting machine learning models in biomedical research, we present our data as AUC, sensitivity, specificity, negative predictive value (NPV), and positive predictive value (PPV) (25). Unlike in medical science, PPV and NPV are not statistical analyses based on the incidence of a certain disease but an internal statistical analysis of the predicted negative or positive value which are truly negative or positive, as used in computer science. As is common practice, we also included results in accuracy to reduce complexity in interpreting our findings. All results are presented with a 95% confidence interval in the tables.

## Results

### Primary Outcome

Results on sensitivity, specificity, NPV, and PPV are presented only for the best model; VGG-16P,. VGG-16P identified acne and rosacea with almost equal success as demonstrated by a specificity and sensitivity of 89.53 and 85.42%, respectively, see Table 2. VGG-16P was successful in distinguishing CTCL from eczema with a low rate of false positives (specificity 84.09%), but it proved more difficult to recognize eczema as seen by VGG-16P reaching a sensitivity rate of 74.29% in CTCL versus eczema. Distinguishing psoriasis from eczema was the task with the lowest performance of the VGG-16P. The best outcome came from identifying eczema, with a sensitivity of 81.79%, but the outcome for identification of psoriasis was inferior, with a specificity of 73.57%.

**Table 2:** Results of best model; VGG-16P.

**Task**	**Specificity**	**Sensitivity**	**PPV**	**NPV**
Psoriasis from Eczema	73.57% (69.76–77.13%)	81.79% (78.51–84.76%)	76.79% (74.18–79.22%)	79.07% (76.03–81.81%)
Acne from Rosacea	89.53% (83.97–93.68%)	85.42% (72.24–93.93%)	69.49% (59.16–78.17%)	95.65% (91.72–97.76%)
CTCL from Eczema	84.09% (80.83–86.99%)	74.29% (67.82–80.05%)	63.16% (58.28–67.78%)	89.90% (87.59–91.83%)

*Result of best model, VGG-16P. The 4 parameters shown are the results in distinguishing psoriasis from eczema, acne from rosacea and CTCL from eczema. The 95% confidence interval is in parenthesis. All results are based on cropped images of the test set*.

### Secondary Outcome

Pre-trained models were superior, as demonstrated by the VGG-16P outperforming the VGG-16N in all analyses. This was further demonstrated by the VGG-16PS being superior to the VGG-16NS with respect to accuracy on all tasks and overall on AUC, see Table 3.

**Table 3:** Performance of all VGG-16 modifications.

	**AUC**	**Accuracy**
**Task 1; Psoriasis vs. Eczema**		
VGG-16P	**86.07% (83.96–88.18%)**	**77.82% (75.35–80.15%)**
VGG-16N	81.74% (79.34–84.14%)	73.70% (71.10–76.18%)
VGG-16PS	83.47% (81.18–85.76%)	74.87% (72.31–77.32%)
VGG-16NS	81.88% (79.49–84.27%)	73.11% (70.49–75.61%)
**Task 2; Acne vs. Rosacea**		
VGG-16P	89.89% (81.98–94.80%)	**88.64% (83.68–92.51%)**
VGG-16N	88.70% (82.36–95.04%)	86.82% (81.62–90.99%)
VGG-16PS	**92.74% (87.54–97.94%)**	88.18% (83.16–92.13%)
VGG-16NS	92.03% (86.60–97.46%)	87.73% (82.65–91.75%)
**Task 3; CTCL vs. Eczema**		
VGG-16P	**88.39% (85.30–91.48%)**	**81.46% (78.55–84.12%)**
VGG-16N	85.55% (82.16–88.94%)	78.90% (75.87–81.71%)
VGG-16PS	86.64% (83.36–89.92%)	78.77% (75.74–81.59%)
VGG-16NS	85.42% (82.02–88.82%)	77.37% (74.27–80.25%)

*Results in AUC and accuracy for all model in all three tasks, best results are highlighted. The 95% confidence interval is in parenthesis. All results are based on cropped images of the test set*.

The VGG-16 architecture without the addition of a STN was slightly superior, as demonstrated by VGG-16P outperforming VGG-16PS, and the VGG-16N and VGG-16NS having similar outcomes.

For all models, the general trend of best performance was on cropped images in the three defined tasks (see Supplementary Table 3), why all results presented in Tables 2, 3 are based on cropped images.

## Discussion

The present retrospective study is an attempt to develop a CAD for more generalized skin diseases that may be of significant help, especially for GPs. The proposed CAD was based on an extensive dataset of clinical images collected from patients consulting a single dermatological department in Denmark. The best performance was obtained by the VGG-16P model when performing the task of distinguishing acne from rosacea (sensitivity 85.42 and specificity 89.53%). Notably, this model distinguished between the diseases on all three tasks with accuracy above 77%, indicating a clinically relevant accuracy compared with the reported diagnostic accuracy in dermatology in general of primary care physicians (48–77%) (5).

Comparing our results to the sensitivity of 67–75% on common multiple-lesion skin diseases reported for the CAD of Liu et al. (12), we find that the present binary model has a potential role in the field of computer-aided diagnostics for common multiple-lesions skin disease. The CAD model of Lui et al. was a multinomial classifier, why further testing of our system in multinomial classification is indicated for true comparison. Head-to-head comparison of suggested CAD models for dermatology seems warranted but it is challenging due to a general lack of online availability (8). To accommodate this paucity, our classification model can be accessed at: https://github.com/anjalilje/Classification-of-skin-diseases.

High performance in distinguishing CTCL from eczema may indicate a yet unidentified usability of CAD tools in diagnosing rare and malignant multiple-lesion skin diseases.

The published image classifiers for single-lesion skin disease show a higher accuracy than our model. Notably, two studies combined have outperformed hundreds of trained dermatologists in identifying melanoma in skin images (9, 10). Accordingly, Esteva et al. achieved an AUC of between 91 and 96%. In comparison, the maximum AUC achieved by our VGG-16PS in distinguishing acne from rosacea was 92.74%. Our best model VGG-16P achieved AUCs between 86.07 and 89.89% for the three defined tasks. The superior performance of CAD models classifying single-lesion skin disease compared to CAD models classifying multiple-lesion skin disease indicates that developing a classification model for generalized dermatology on non-standardized imagery may prove to be a more complex task.

Overfitting is a risk in the present study for several reasons: Firstly, acne, rosacea and CTCL were represented only by small datasets, and balancing the data did enhance the classification performance on these three skin diseases, thereby confirming a level of overfitting. Still, the performance enhancement on balanced datasets was only slightly superior to that of unbalanced datasets. Secondly, overfitting due to selection bias is a potential problem of CNN models in dermatology (26). The present retrospective study may therefore suffer from selection bias.

Wu et al. showed that multi-lesion skin disease can be classified at the level of single-lesion skin disease, on highly selected image material (13).

Our study was conducted on a clinical image database, we argue this to be less prone to selection bias, due to our content originating from clinical photographers, patients, and clinicians in non-specific clinical situations.

To which extend the results of this study can be extrapolated to clinical use, could be further investigated by head to head testing of the CAD model and trained physicians, like the man and machine approach from a recent study in single-lesion skin disease classification (15). But there is a need for designs of real-time clinical intervention studies for true estimates of the clinical diagnostic accuracy of CAD models not based on dermatoscopic images. This paucity of prospective clinical tests in the development of CADs in dermatology has been criticized (27, 28). Fourthly, no quantification of unknown biases was conducted, this represents a limitation, as an example a certain diseases may be represented by clinical photography to a higher degree than others. Unknown biases could be tested on an external dataset. Since no external dataset are available for the selected disease categories, this further argues for making CAD models available for online testing.

And finally, the grouping of several ICD-10 codes into major disease categories may result in overfitting, since some subtypes of diseases have less similar morphology than others. Thus, overall, the level of overfitting is considered to be of minor importance.

One limitation of this study was the contents, which comprised both healthy skin images and non-skin images. Non-skin images were cleansed successfully with a 99% accuracy, why the effect of their inclusion was minute. Healthy skin images were estimated to comprise 3.7% of the material in the sampling, which may have had a negative effect on the performance outcome. Hence, the true performance of the models may have been underestimated. Another limitation is racial bias, as the data source consisted primarily of Fitzpatrick skin type II-III patients. Concerns have been raised of racial bias in CAD in dermatology because databases used for machine learning have historically had an overrepresentation of Caucasian data (29).

Grouping ICD-10 codes into major disease categories may not only represent a limitation but could also be considered a strength in our study. Disease categories increase the amount of data, thus enhancing the performance of the models. Moreover, disease categories represent a simpler outcome and may therefore be more clinically relevant for a GP, as the primary purpose of CAD in general dermatology should be to assist correct and early diagnosing, treatment, and triaging.

ICD-10 coding of the images were considered as high quality categorization of the images due to two factors. Firstly, the ICD-10 codes were provided by a physicians employed at the dermatological department of a University Hospital. Secondly, all ICD-10 codes related to the only rare disease included, CTCL, were based on histological verification.

Images used for single-lesion disease classification like malignant melanoma are often taken by highly standardized methods (8). However, CNN models like the present one trained on various types of images with varying quality may perform better in real-life usage. Most dermatological diseases have a more generalized skin manifestation than malignant melanoma, and the sparsity of dermatoscopes in the primary sector is also a limitation (20).

The results obtained in this study are encouraging. Medical students, resident doctors, and GPs with little to no training in the field of dermatology have been shown to perform very poorly in diagnosing dermatological diseases (19) and may benefit from a CAD model performing to the present level.

Furthermore, our findings support that in the future of all dermatological diagnostics, man and machine together will very likely be superior to man alone as seen for CAD models developed for single-lesion skin disease (15). Even so, implementing CAD models in dermatology should be accommodated by thorough prospective clinical testing to ensure true estimates, thus ensuring patient safety, efficacy, and effectiveness.

## Data Availability Statement

The datasets presented in this article are not readily available because the datasets consist of clinical images of patients with skin disease, which cannot be shared in accordance to the European General Data Protection Regulation. The skin disease classification algorithm is available only at https://github.com/anjalilje/Classification-of-skin-diseases.

## Ethics Statement

The studies involving human participants were reviewed and approved by The Regional Ethics Committee of Central Denmark Region. Written informed consent from the participants' legal guardian/next of kin was not required to participate in this study in accordance with the national legislation and the institutional requirements.

## Author Contributions

KT: main contributor to all aspects of the manuscript. AC: software developer, significant contributor to the methods and results sections of the manuscript, and designer of figures of software architecture. LI: main supervisor in the clinical aspects of the manuscript and significant contributions to the introduction and discussion. HL: co-supervisor in the clinical aspects of the manuscript, large contributions especially to the introduction, discussion and to table content. OW: supervisor of software development and significant contributor to all aspects of the manuscript.

## Conflict of Interest

The authors declare that the research was conducted in the absence of any commercial or financial relationships that could be construed as a potential conflict of interest.

## Abbreviations

AUH: Aarhus University Hospital
AUC: Area under the curve
CAD: Computer aided diagnostic
CNN: Convolutional neural network
CTCL: Cutaneous t-cell lymphoma
GP: General practitioner
ILSVRC: ImageNet Large Scale Visual Recognition Challenge
ICD-10: International Classification of Diseases, 10th Version
NPV: Negative predictive value
PPV: Positive predictive value
STN: Spatial transmitter network
VGG: Visual Geometry Group.
