# Supplementary material for: Deep Learning for Diagnostic Binary Classification of Multiple-Lesion Skin Diseases
Source: Front Med (Lausanne). 2020 Sep 22;7:574329. doi: 10.3389/fmed.2020.574329 (PMC7536339; doi:10.3389/fmed.2020.574329)
Supplement: Supplementary file 1 [file Presentation_1.PDF]

Supplementary material

Content:

Online only figure: 2

Online only table: 3

Supplementary figure 1: Data-cleanser

Supplementary figure 2: Image cropping

Supplementary table 1: ICD-10 codes distributed in disease categories

Supplementary table 2: Hyperparameters and hardware specification

Supplementary table 3: Data augmentation performance

Supplementary figure 1: Data-cleanser

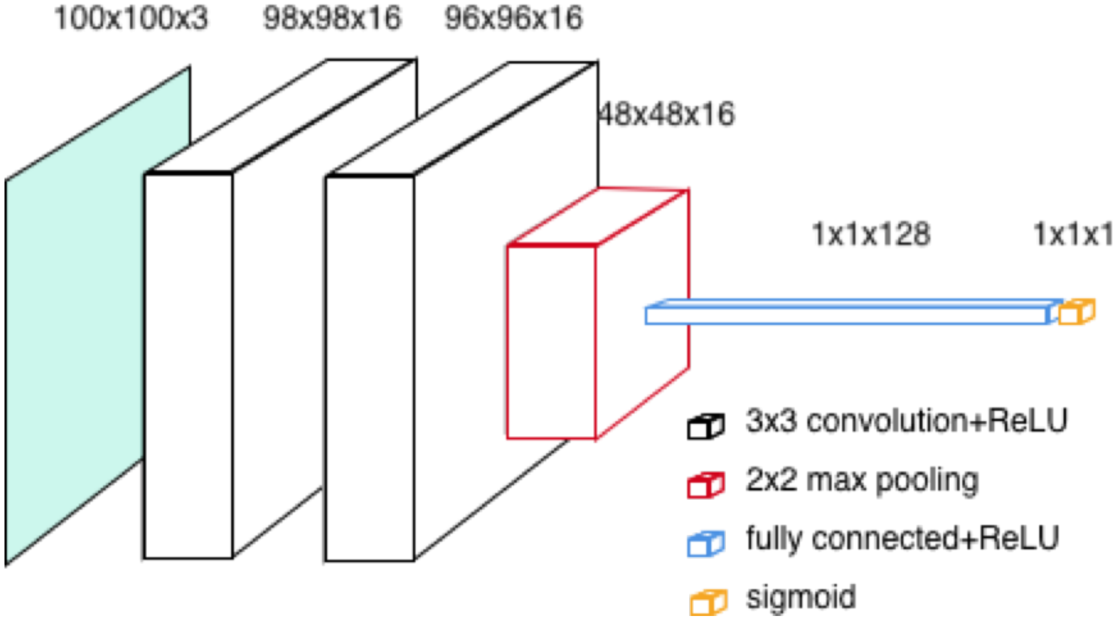

A simple binary CNN model was built to remove yellow identification slips and other non-skin images. This model was trained on 200 non-skin images and 200 skin images converted to the Red Green Blue (RGB) colour space. The model was tested on 300 images; 150 from each class, and achieved a 99% accuracy in identifying non-skin images. 3,098 non-skin images were removed by this method, leaving a total of 16,543 images that were used. See Table 2 for the data distribution by disease category before and after data-cleansing.

Supplementary figure 2: Image cropping

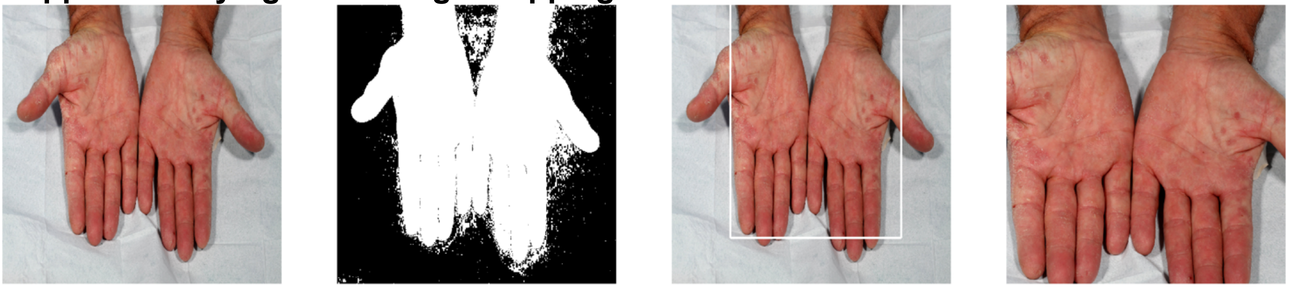

Examples of image cropping, from the left original image file, two images in the middle column displaying a focus on the diseased skin, and finally the right column displaying the focused images after cropping.

Supplementary table 1: ICD-10 codes distributed in disease categories

Disease	ICD-10 (nPatients)
Psoriasis	DL400 (752), DL400C (2), DL404 (25), DL408 (3) and DL409 (8)
Eczema	DL200 (187), DL208 (3), DL 208A (45), DL 208B (3), DL208D (3), DL 209 (28), DL 300 (46), DL308C (24) and DL309 (531)
CTCL	DC840 (131), DC840A (2), DC841 (6), DC844 (2) and DC845 (16)
Acne	DL700 (58), DL701 (13), DL705 (2), DL708 (3), DL708C (5) and DL709 (50)
Rosacea	DL710 (20), DL711 (150), DL718A (12), DL718C (7), DL718D (2), DL718 (67) and DL719 (136)

ICD-10 code distribution in disease categories of the Aarhus University Hospital dataset, number of patients of each ICD-10 code is in parenthesis.

Supplementary table 2: Hyperparameters and hardware specification

	VGG-16P	VGG-16N	VGG-16PS	VGG-16NS
**Hyperparameters**				
Input size	250 x 250 x 3	250 x 250 x 3	Localisation: 150 x 150 x 3 VGG16: 224 x 224 x 3	Localisation: 150 x 150 x 3 VGG16: 224 x 224 x 3
Epochs	O: 90 C: 80 OB: 70 CB: 80	O: 110 C: 120 OB: 120 CB: 100	O: 120 C: 120 OB: 90 CB: 110	O: 80 C: 110 OB: 110 CB: 100
Batch size	64	64	64	64
No. of Parameters	Total: 14 753 859 Trainable: 7 118 595 Non-trainable: 7 635 264	Total: 14 753 859 Trainable: 14 753 859 Non-trainable: 0	Total: 14 838 185 Trainable: 7 202 921 Non-trainable: 7 635 264	Total: 14 838 185 Trainable: 14 838 185 Non-trainable: 0
Learning rate	Initial: 10^{-4}	Initial: 10^{-4}	Initial: 10^{-4}	Initial: 10^{-4}
Dropout rate	0.5	0.5	0.5	0.5
L_2 penalty	5 x 10^{-4}	5 x 10^{-4}	5 x 10^{-4}	5 x 10^{-4}
Gradient clipping	1.0 by norm	1.0 by norm	1.0 by norm	1.0 by norm
Coding language	Python	Python	Python	Python
Software library	Tensorflow	Tensorflow	Tensorflow	Tensorflow
**Hardware specification**				
No. GPU clusters	GPU Ram	Host Ram	GPU architecture	Host CPU
7-8	11-12 GB	256 GB	Volta, Maxwell, Turing, Pascal and Kepler Pascal	2 x Xeon E5-2620 v4 8c/16t - 2.1 GHz

Hyperparameters and hardware specification related to each of the four models. Original data (O), cropped data (C), balanced original (OB) and balanced cropped data (CB).

Supplementary table 3: Data augmentation performance

	VGG-16P		VGG-16N		VGG-16PS		VGG-16NS	
**Combined**	Result	Data type	Result	Data type	Result	Data type	Result	Data type
Accuracy	**80.20%**	C	76.87%	C	77.80%	C	76.09%	C
**Task 1; psoriasis versus eczema**								
Accuracy	**77.82%**	C	73.70%	C	74.87%	C	73.11%	C
AUC	**86.07%**	C	81.74%	C	83.47%	C	81.88%	C
**Task 2; acne versus rosacea**								
Accuracy	88.64%	C	86.82%	C	**90.00%**	CB	87.73%	C
AUC	92.67%	CB	90.98%	OB	**92.74%**	C	92.03%	C
**Task 3; CTCL versus eczema**								
Accuracy	**82.10%**	OB	81.71%	O	80.95%	O	79.41%	O
AUC	**88.84%**	OB	87.80%	OB	87.18%	CB	88.30%	OB

The best performance of each task including data augmentation type. The combined accuracy of each model on all three tasks is included. The best model for each task is highlighted. Data abbreviations: original (O), original balanced (OB), cropped (C) and cropped balanced (CB).
